# Aperiodic Modulation of Graphene Driven by Oxygen-Induced Reconstruction of Rh(110)

**DOI:** 10.1021/acs.jpcc.3c02643

**Published:** 2023-08-30

**Authors:** Haojie Guo, Mariano D. Jiménez-Sánchez, Enrique G. Michel, Antonio J. Martínez-Galera, José M. Gómez-Rodríguez

**Affiliations:** † Departamento de Física de la Materia Condensada, Universidad Autónoma de Madrid, E-28049 Madrid, Spain; ‡ Instituto Nicolás Cabrera, Universidad Autónoma de Madrid, E-28049 Madrid, Spain; § Condensed Matter Physics Center (IFIMAC), Universidad Autónoma de Madrid, E-28049 Madrid, Spain; ∥ Departamento de Física de Materiales, Universidad Autónoma de Madrid, E-28049 Madrid, Spain

## Abstract

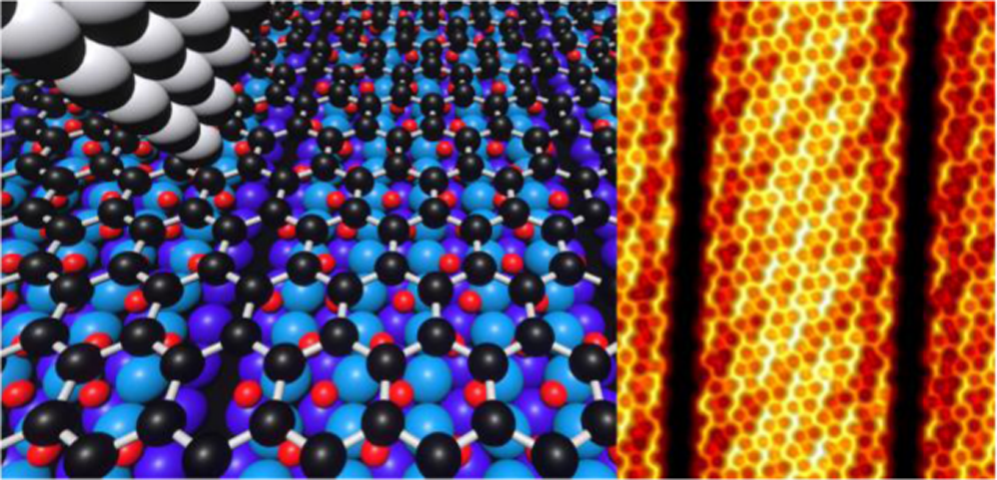

Artificial nanostructuring of graphene has served as a platform to induce variations in its structural and electronic properties, fostering the experimental observation of a wide and fascinating phenomenology. Here, we present an approach to graphene tuning, based on Rh(110) surface reconstruction induced by oxygen atoms intercalation. The resulting nanostructured graphene has been characterized by scanning tunneling microscopy (STM) complemented by low-energy electron microscopy (LEEM), micro low-energy electron diffraction (μ-LEED), micro angle-resolved photoemission spectroscopy (μ-ARPES), and micro X-ray photoelectron spectroscopy (μ-XPS) measurements under ultrahigh vacuum (UHV) conditions at room temperature (RT). It is found that by fine-tuning the O_2_ exposure amount, a mixture of missing row surface reconstructions of the metal surface below the graphene layer can be induced. This atomic rearrangement under the graphene layer results in aperiodic patterning of the two-dimensional (2D) material. The electronic structure of the resulting nanostructured graphene is dominated by a linear dispersion of the Dirac quasiparticles, characteristic of its free-standing state but with a *p*-doping character. The local effects of the underlying missing rows on the interfacial chemistry and on the quasiparticle scattering processes in graphene are studied using atomically resolved STM images. The possibilities offered by this nanostructuring approach, which consists in inducing surface reconstructions under graphene, could provide a novel tuning strategy for this 2D material.

## 1 Introduction

More than 15 years after the first isolation of graphene,^1^ the continuous observation of a fascinating new phenomenology,^2−5^ derived from the unique behavior of the quasiparticles in this precursor of the two-dimensional (2D) materials research, does not stop. Initially, the work focused on pristine graphene layers, where phenomena such as the massless quasiparticle behavior,^6^ resulting in a linear band structure, or quantum hall effect,^7,8^ have been observed. Interestingly, the two-dimensional nature of this material makes it more sensitive to the presence of defects, as well as to other elements placed in the local environment. This fact has opened up new research opportunities, providing a platform for the controlled tuning of graphene properties to pursue the exploration of novel phenomena.

The presence of various defects such as C vacancies^9^ or graphene bubbles^10^ has been shown to induce an interesting phenomenology. While for the former, the existence of localized states, characterized by a certain magnetic moment,^9^ has been reported, for the latter, the existence of giant pseudomagnetic fields has been established.^10^ From the point of view offered by the possibility of tuning the properties of graphene by interacting with other elements, the deposition of atoms,^11^ molecules,^12^ and clusters^13^ on top of graphene has been shown to induce, for example, magnetism,^11^ doping,^12^ bandgap opening,^13^ or asymmetries in the group velocities of quasiparticles^13^ among others. Accordingly, the choice of support has also provided a versatile tool to modify the properties of graphene. These effects range from only slight changes in the electronic properties, such as doping^14^ or the opening of minigaps^15^ and the formation of band replicas,^15^ due to the potential felt by the quasiparticles, associated with the superstructures resulting from the lattice mismatch between graphene and the support, known as moiré patterns, to the complete absence of Dirac cones near the Fermi level.^16,17^ Both the metal element forming the support and the symmetry of its atomic arrangement have been shown to be key parameters to control the properties of graphene by inducing all this phenomenology.^18^ Taking these ideas a step further, other approaches, such as adding different elements beneath graphene as another layer of this material or an intercalation layer at its interface with the substrate, have also been explored. Interestingly, the addition of a second layer led to the observation of unconventional Cooper pairing in twisted bilayers, where both graphene sheets are rotated under the so-called magic angle.^19^ Likewise, the incorporation of an intercalation layer has induced effects ranging from doping, which can be tuned by the electronegativity of the intercalant,^20,21^ to the recovery of Dirac cones in cases in which they had been suppressed by the interaction of graphene with the support.^22,23^

Surface reconstruction is a phenomenon where a few atoms of the outermost layers suffer lateral displacements with respect to their original positions or undergo a complete transformation of their periodicity with respect to the bulk.^24,25^ Reconstruction of the surface atomic arrangement is very common in semiconductor materials and less so in metals due to the absence of dangling bonds that increase the surface free energy.^25^ However, an unreconstructed bare metal can still experience a surface reconstruction due to the adsorption of specific atoms and molecules.^24,26^ In the present work, this circumstance is exploited to modify graphene by inducing a surface reconstruction underneath through intercalation, thus taking a step forward in the development of approaches to tune this 2D material. In particular, a nanostructured graphene system is obtained by O intercalation between this 2D material and a Rh(110) surface. This metal support was chosen because the chemisorption of oxygen atoms on it induces a surface reconstruction in the form of missing rows to reduce the stress caused by the repulsive adatom–adatom interaction.^27−30^ The combination of different characterization methods has revealed the structural and electronic properties of the resulting nanostructured graphene, as well as the local effects of the missing rows underneath, both in the interfacial chemistry and in quasiparticle scattering. This work may pave the way for future investigations, related to the tuning of the properties of graphene or other 2D materials on metals, by taking advantage of the reconstruction of the metal surface upon intercalation of different elements.

## 2 Experimental Methods

### 2.1 Growth of Graphene on Rh(110)

Monolayers of graphene on Rh(110) were grown in situ by chemical vapor deposition under ultrahigh vacuum (UHV) conditions, using ethylene (C_2_H_4_) as the precursor molecule. To this end, the Rh(110) single crystal was cleaned through cycles of Ar^+^ sputtering at 1 keV, followed by annealing at 950 °C at an oxygen partial pressure of 2 × 10^–6^ Torr, and finished with a flash-annealing in UHV at 950 °C and 1050 °C. Ethylene exposure was then performed by opening a leak valve, connected to a high-purity source of this gas, at a pressure of 3 × 10^–7^ Torr for 150 s, while maintaining the Rh(110) substrate at 900 °C.

For low-energy electron microscopy (LEEM), micro low-energy electron diffraction (μ-LEED), micro angle-resolved photoemission spectroscopy (μ-ARPES), and micro X-ray photoelectron spectroscopy (μ-XPS) experiments, a different approach to graphene growth was used to avoid the contribution of different rotational domains of graphene on Rh(110) to the total signal.^18^ For this purpose, the metal surface was exposed to ethylene at high temperatures, allowing its thermal decomposition and the dissolution of C atoms into the bulk. Then, by keeping the substrate at a lower temperature, single isolated islands of graphene were formed by C atom segregation from the substrate. All this phenomenology was monitored in situ by LEEM.

Sample temperature was measured in both experimental systems by using an infrared digital pyrometer.

### 2.2 Intercalation of Oxygen

Oxygen intercalation at the graphene/Rh(110) interface was achieved by maintaining the sample at 320 °C during its exposure to molecular oxygen (O_2_) at a partial pressure of 1 × 10^–6^ Torr. In scanning tunneling microscopy (STM) experiments, where a monolayer of graphene covered the entire Rh(110) surface, 3.6 × 10^3^ L was typically required to achieve an intercalated coverage of ≈0.4 ML, while during the experiments at the Nanospectroscopy beamline, the dosage used was much lower (900 L) since the isolated graphene islands coexist with nearby bare metal regions. This difference in the procedure can be understood as follows: the presence of a fraction of bare Rh(110) surface directly supports the dissociation of molecular oxygen, allowing the intercalation process. On the contrary, thermally activated etching^31^ of the graphene layer is required to create bare Rh(110) regions, where O_2_ molecules can dissociate, fostering the intercalation of the resulting O adatoms at the interface between graphene and Rh in neighboring regions.

### 2.3 STM Measurements

STM experiments were performed using a home-built variable temperature scanning tunneling microscope (VT-STM),^32,33^ placed in a UHV chamber (base pressure below 10^–10^ Torr) connected to the adjacent preparation chamber and delimited by a gate valve. All experimental data were measured at room temperature (RT) in the constant current mode, using electrochemically etched W tips and with the bias voltage applied to the sample. Data were acquired and post-analyzed using the WSxM software.^34^

### 2.4 LEEM, μ-LEED, μ-ARPES, μ-XPS Measurements

These techniques were executed using the spectroscopic photoemission and low-energy electron microscope (SPELEEM),^35,36^ at the Nanospectroscopy beamline of the Elettra Sincrotrone Trieste Laboratory. This instrument combines imaging, diffraction, and spectroscopy operation modes under UHV conditions, using either electrons or photons as probe sources. The kinetic energy of the electrons emitted or scattered electrons by the sample was controlled by the bias voltage applied to the sample, known as the start voltage. For LEEM measurements, only the zero-order diffraction beam was used for imaging (Bright Field). For μ-LEED, ARPES, and XPS data, the imaging area was limited to a diameter of less than 2 μm using illumination or a field-limiting aperture. The monochromated soft X-ray beam was incident on the sample at a grazing angle of 16° from the surface plane.^35,36^ The energy resolution of Nanospectroscopy beamtime for spectroscopic data is below 100 meV. ARPES data were collected by acquiring constant-energy surfaces below the Fermi level, with an energy step-size of 0.025 eV, and subsequently stacked upon each other. Then, the band structure can be obtained by cutting along the desired high symmetry directions. All measurements were performed at RT.

## 3 Results and Discussion

**Figure 1:**
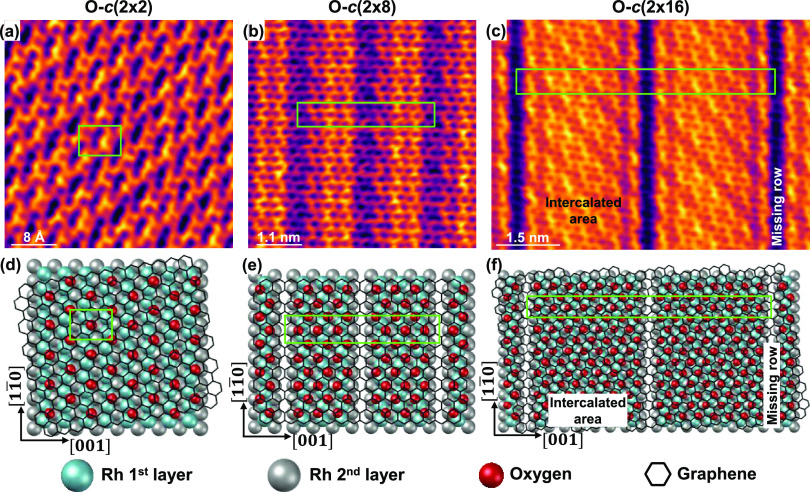
Oxygen phases below graphene with respect to Rh(110). (a–c) STM topography images of O-*c*(2 × 2) without Rh(110) substrate reconstruction, O-*c*(2 × 8) with (1 × 4) substrate reconstruction as missing row and O-*c*(2 × 16) with (1 × 8) substrate reconstruction as missing row, respectively. (d–f) Schematic of a plausible atomic arrangement for each oxygen structure shown in panels (a–c). The green rectangles indicate the unit cell. Tunneling parameters: (a) *V*_s_ = 0.1 V; I_t_ = 17.5 nA; size: 4 × 4 nm^2^. (b) *V*_s_ = −60 mV; *I*_t_ = 1 nA; size: 5.5 × 5.5 nm^2^. (c) *V*_s_ = 0.25 V; *I*_t_ = 5.2 nA; size: 7.5 × 5.5 nm^2^.

Figure 1 outlines some of the observed superstructures formed via O intercalation at the interface between graphene and the Rh(110) surface. The periodicities of these sandwiched superstructures is related to the structural parameters describing the unit cell of the unreconstructed Rh(110) surface. Figure 1a shows an atomic resolution STM image displaying the graphene surface along with protrusions, whose periodicity is consistent with a *c*(2 × 2) structure with respect to Rh(110). Meanwhile, Figure 1b,c shows two STM images illustrating a completely different scenario. As it can be observed, regularly equispaced straight-line regions, imaged as depressions, appear superimposed with graphene atomic resolution. The repetition periods of these lines observed in Figure 1b,c are (1 × 4) and (1 × 8), respectively, with respect to Rh(110).

To understand the features observed in Figure 1a–c, a brief review of previously reported data on the structural properties of the superstructures resulting from the dissociative O chemisorption on Rh(110) surfaces is necessary. Briefly, for oxygen coverages of 0.5, 0.67, 0.75, and 0.8 ML, respectively, the Rh(110) surface undergoes (1 × 2), (1 × 3), (1 × 4), and (1 × 5) reconstructions as missing rows, yielding (2 × 2)*p*2*mg*, *c*(2 × 6), *c*(2 × 8), and *c*(2 × 10) O/Rh(110) superstructures.^29,30^ These types of (1 × *n*) surface reconstructions imply that one surface closed-packed row along the [11̅0] direction is missing out of *n* atomic spacings along the [001] one, and no oxygen atoms are located along the resulting stripes. Considering this, the superstructure observed in Figure 1a could be interpreted as an O-*c*(2 × 2) phase on Rh(110) under the graphene cover, which does not involve substrate reconstruction. In contrast, the stripe patterns in Figure 1b,c could be attributed to the reconstruction of the substrate Rh(110) in the form of missing rows, resulting in the formation of O-*c*(2 × 8) and O-*c*(2 × 16) structures, respectively. Moreover, by using the oxygen arrangement as a fingerprint of the Rh(110) crystallographic direction, information about the graphene rotation angle was also derived. Figure 1a,c would correspond to a twist angle between graphene and the Rh[001] direction of about 10°, while in the case of Figure 1b, they would be aligned or nearly aligned. Orientation configurations around these ones are preferred when graphene is grown on Rh(110).^18^ It should also be noted that the continuity of the graphene monolayer is not interrupted by the underlying missing row reconstruction of the substrate (see Figures 1b,c and S1), although the sandwiched superstructure shows a lower apparent height in the missing row areas (with a total corrugation of ≈35 pm). In this sense, the graphene in these regions seems to behave as a continuous rippling sheet across the surface.

To illustrate the above situations, a plausible schematic representation of the atomic arrangement is proposed in Figure 1d–f for each oxygen phase shown in Figure 1a–c, respectively, where the green rectangles mark the corresponding unit cell. In this framework, oxygen adatoms are positioned on the threefold fcc-hollow sites coordinated by two surface Rh atoms and another one from the second layer.^27−29^ Furthermore, two distinct areas of graphene can be found when oxygen induces substrate reconstruction: the intercalated area (IA) or the missing row (MRA) area, as labeled in Figure 1f.

The above comparison with the case of O on Rh(110) inevitably raises the question of whether or not the graphene cover plays a role in the underlying atomic arrangement. Compared to the O-(2 × 2)*p*2*mg* arrangement reported on the bare metal, where the oxygen atoms induce a (1 × 2) missing row reconstruction of the Rh(110) substrate, here the O-*c*(2 × 2) does not involve any Rh(110) surface reconstruction under graphene. Likewise, O-*c*(2 × 16) with (1 × 8) surface Rh(110) reconstruction also differs from the available literature for the case of O/Rh(110), since the maximum spacing in the missing row reconstruction of Rh(110) was reported to be (1 × 5). Therefore, it seems that the graphene layer induces changes in the chemisorption of oxygen on Rh(110). Such an idea was also conceived in the Gr/O/Ir(111) system,^37^ where new oxygen phases under graphene, which do not exist on the bare Ir(111) metal, were reported. A possible explanation for the observed experimental evidences is given in the following lines: O adatoms chemisorbed on Rh(110) are negatively charged due to electron transfer from Rh atoms to the more electronegative O ones. As a result, there is a repulsive adatom–adatom interaction, which induces stress in the Rh(110) surface, given the fact the O atoms are bounded to Rh ones in the topmost surface layer. This stress seems to be relaxed via the formation of missing row reconstructions in the substrate. Nevertheless, when a graphene cover is over the O adatoms, the atomic lattice of this 2D material could receive some of the stress, which otherwise would be completely induced in the underlying Rh(110) support. It could explain, for instance, why a missing row reconstruction is not needed at low amounts of O intercalation in the case of the O-*c*(2 × 2) superstructure.

**Figure 2:**
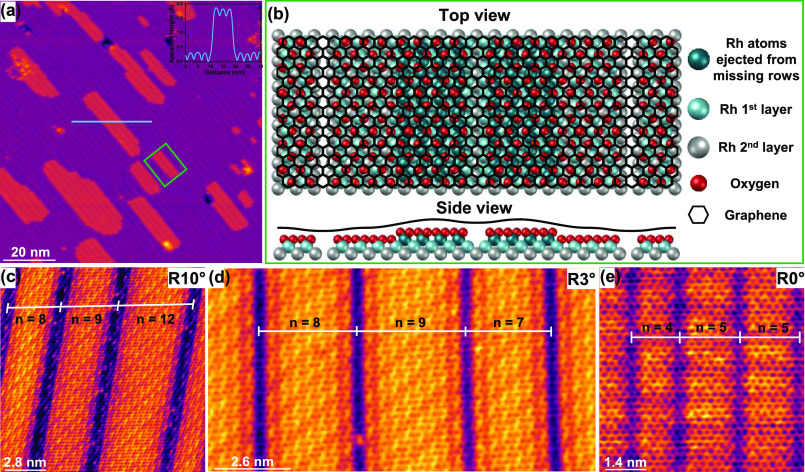
Randomly distributed missing row reconstruction periodicity of Rh(110). (a) Large-scale STM image showing the general morphology of the substrate Gr/Rh(110) after oxygen intercalation in a region where oxygen induces substrate reconstruction. Elongated islands with different dimensions coexist with planar regions, both featuring the same characteristics. Inset shows the apparent height profile along the blue line. (b) Schematic representation describing the features observed within the green rectangle sketched in panel (a). Rh atoms ejected from the missing row areas aggregate together to form monoatomic step-height islands of Rh(110). (c–e) Set of STM images displaying the plurality of the Rh(110) missing row reconstruction periodicities induced by oxygen below different twist angles of graphene. Tunneling parameters: (a) *V*_s_ = 2 V; *I*_t_ = 0.3 nA; size: 100 × 100 nm^2^. (c) *V*_s_ = 50 mV; *I*_t_ = 18.1 nA; size: 14 × 14 nm^2^. (d) *V*_s_ = 0.4 V; *I*_t_ = 10.3 nA; size: 13 × 6.8 nm^2^. (e) *V*_s_ = 0.1 V; *I*_t_ = 10.0 nA; size: 7 × 7 nm^2^.

The sandwich superstructures involving substrate reconstruction were those found predominantly under the intercalation parameters used in the present work. A broader perspective can be gained by looking more closely at the regions where these oxygen-induced atomic rearrangements of Rh(110) occur. Figure 2a shows a large-scale STM image of an oxygen-intercalated region with Rh(110) substrate reconstruction. As observed, flat zones coincide with elongated islands of different dimensions, both sharing the same features: 1D channels (substrate reconstruction), shown as depressions, permeating the entire region. The profile along the blue line in Figure 2a, which is shown in the inset, proves that these islands have an apparent height of ≈1.5 Å. A plausible atomic scale configuration is sketched in Figure 2b, which depicts the features within the green rectangle indicated in Figure 2a, as will be discussed later. Figure 2c–e shows a series of STM images of three rotational domains of graphene on Rh(110) intercalated by oxygen, where different substrates’ missing row reconstruction periodicities, labeled in each panel, coexist in the same region.

The measured apparent height of the islands is in agreement with the single-step height of Rh(110), suggesting that they are formed by Rh atoms in the same configuration as in the (110) plane of bulk Rh. Moreover, the presence of missing rows also indicates the chemisorption of oxygen atoms onto Rh(110) within the islands. These two observations could be rationalized in terms of a rearrangement of Rh atoms ejected from the missing row areas after the adsorption of oxygen, forcing the former to aggregate together to form new terraces on the Rh(110) facet with the same rectangular configuration. Analogous phenomena have been observed for O on bare Rh(110),^38,39^ as well as after the adsorption of various species on other metal surfaces, such as acetate molecules on Au(110)^26^ or Si on Ag(110).^40^ Furthermore, in the present case, all these processes must take place at the interface between graphene and Rh(110) since experimental STM data showed that the graphene monolayer was not disrupted between the planar regions and the new islands (see Figure S2). This also proves the strength and stability of graphene to handle such complex atomic rearrangements below. The above description is illustrated by the scheme shown in Figure 2b.

As already can be seen in Figure 2a, and further demonstrated in Figure 2c–e, the (1 × *n*) periodicity of the missing row reconstruction of Rh(110) seems to follow a random distribution over the entire surface, ranging from *n* = 4 to *n* = 12. Therefore, the O-*c*(2 × 8) and O-*c*(2 × 16) phases described in Figure 1 and any other O-*c*(2 × 2*n*), with *n* = [4–12], are found only locally, and the surface is characterized by a long-range aperiodicity. In other words, in this case, graphene could be considered as a one-atom-thick sheet of carbon atoms that ripples aperiodically over the entire surface. Based on the above results, it could be pointed out that the formation of the missing rows across the sandwiched superstructures would have a certain stochastic character due to the fact that the different (1 × *n*) missing rows, with 12 > *n* > 4, are thermodynamically allowed. Nevertheless, it seems that there must be some interaction between the missing rows since no (1 × *n*) rearrangements for *n* < 4 are found. Furthermore, it cannot be excluded that the distribution of the missing rows along the [001] direction of Rh could be influenced by some defects that could be created during the growth of graphene or the intercalation of oxygen. In addition, no experimental evidence was found for a possible effect of the rotational domains of graphene on the periodicity of the missing row reconstruction.

**Figure 3:**
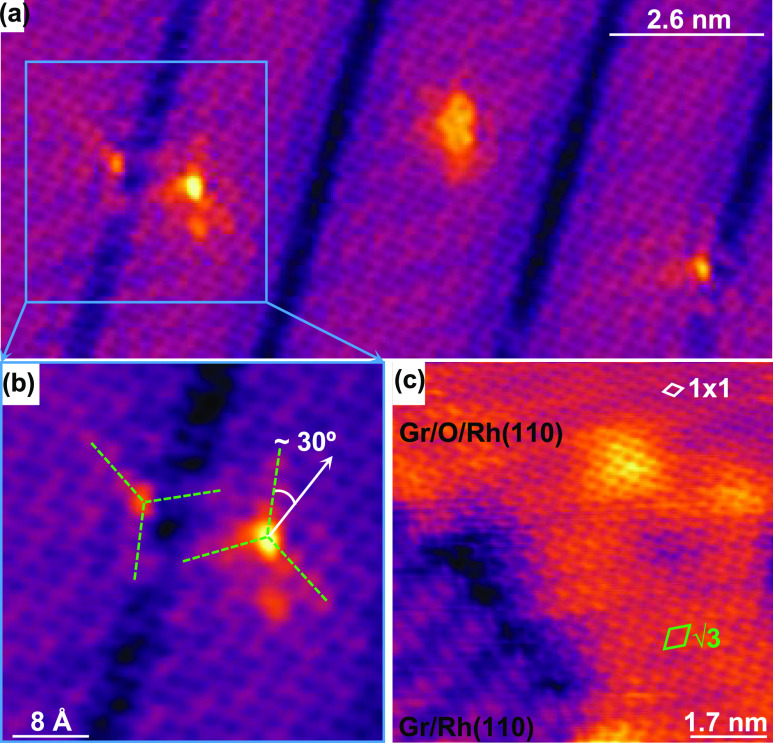
Existence of graphene quasiparticle scattering in oxygen-intercalated Gr/Rh(110). (a) STM image acquired in a region where different defects induce the appearance of a modulation pattern in the topography of graphene overlayer. (b) Zoom-in image within the area indicated by the blue square in (a). Bright arms (green dashed lines) around the defects are identified as the threefold intervalley scattering directions of graphene quasiparticles. (c) STM image showing the absence of quasiparticle interference on the bare Gr/Rh(110) region, in sharp contrast with the neighbor oxygen-intercalated area. Tunneling parameters: (a) *V*_s_ = 0.4 V; *I*_t_ = 10.3 nA; size: 13 × 6 nm^2^. (b) *V*_s_ = 0.4 V; *I*_t_ = 10.3 nA; size: 4 × 4 nm^2^. (c) *V*_s_ = 70 mV; *I*_t_ = 1.5 nA; size: 8.5 × 8.5 nm^2^.

Once the structural properties of the sandwiched Gr/O/Rh(110) nanostructures were established, the electronic properties of the system were also investigated. Such studies have been focused on regions where oxygen induces substrate reconstruction under graphene since these are the most commonly found. Figure 3a shows an STM topography of Gr/O/Rh(110), where the graphene lattice is aligned, or nearly aligned, with the Rh[001] direction, with various point defects. As can be seen, an additional modulation pattern is generated and superimposed on the graphene lattice in the vicinity of the defects. A magnified STM image in the region bounded by the blue square in Figure 3a is shown in Figure 3b. Three protruding arms (marked by green dashed lines), forming an angle of ≈30° with respect to the graphene lattice, can be distinguished around the two defects present in this region. In both cases, the protruding arms are superimposed by a periodic modulation with (√3 × √3)-R30° periodicity with respect to the graphene lattice. Moreover, from STM images such as the one shown in Figure 3c, which depicts the boundary between two regions of pristine and oxygen-intercalated Gr/Rh(110), it can be deduced that such modulation is present near the boundary, within the Gr/O/Rh(110) region, but not in the pristine region.

A direct explanation for the origin of these patterns, observed in the STM images of Figure 3, could be related to the scattering of graphene quasiparticles by defects or impurities. In particular, in ideal graphene or well-decoupled graphene/substrate systems, the intervalley scattering processes are translated into short-wavelength modulations of the Local Density of States (LDOS) with (√3 × √3)-R30° periodicity with respect to the graphene lattice, exhibiting three protruding arms along the high symmetry directions of the pattern.^9,14,41,42^ Thus, the features observed in Figure 3 could be related to intervalley scattering processes of the Dirac quasiparticles in the graphene layer due to defects or impurities. It evidences that the graphene layer is efficiently decoupled from the Rh(110) substrate, which is even more emphasized since such scattering processes do not occur on bare Gr/Rh(110). However, the shorter extension of the perturbations, compared to other systems such as graphene on SiC, and HOPG,^9,41,42^ could also indicate a higher level of graphene interaction in the present case with O/Rh(110) underneath. Furthermore, despite the rippling character of graphene induced by the presence of both IA and MRA below, the fact that such patterns can be imaged in these two regions (see also Figure S3) suggests that the graphene monolayer does not exhibit dissimilar electronic properties in these regions. This could be tentatively explained by the fact that the intercalation of oxygen atoms on the IA might be sufficient to allow the decoupling of the entire graphene sheet from the Rh(110) surface in this specific region and also in the MRA. It is noteworthy to mention that intervalley scattering patterns, similar to those observed in Figure 3c at the boundary between intercalated and non-intercalated regions, have also been reported to be present in the vicinity of graphene edges in weakly coupled systems.^43^ It appears that although the graphene layer is not interrupted across these boundaries, they induce quasiparticle scattering processes that are not produced by the MRA.

**Figure 4:**
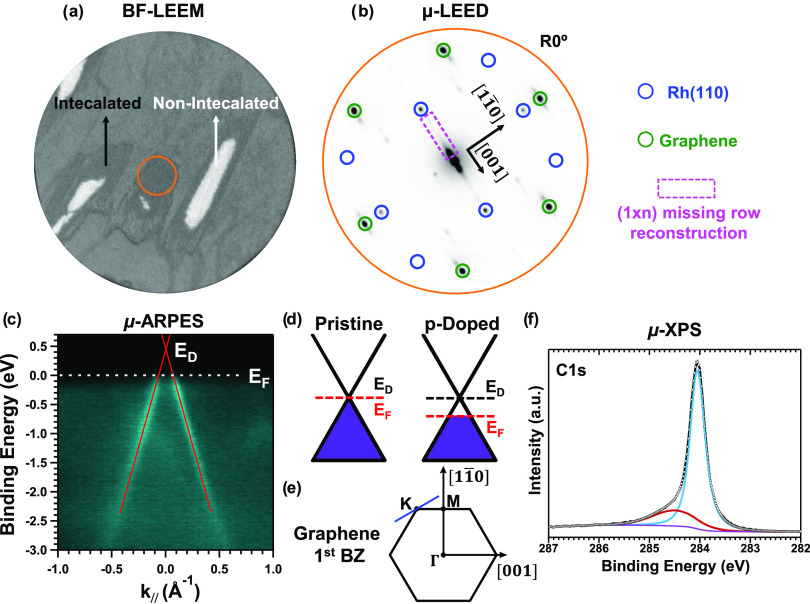
Electronic features on the aligned domain (R0°) of Gr/O/Rh(110) proved at the μ-scale using imaging microscopy, diffraction, and spectroscopy techniques. (a) Bright-field (BF) LEEM image showing oxygen-intercalated graphene islands grown on Rh(110) as well as non-intercalated Gr/Rh(110). The field of view is 10 μm, and the electron energy is 10 eV. (b) Corresponding μ-LEED pattern recorded, with an electron energy of 45 eV, in the area indicated by the orange circle in panel (a) revealing a R0° graphene. (c) μ-ARPES intensity plot of Gr/O/Rh(110) along the perpendicular direction of Γ–*K* (see the blue line in panel (e)) acquired in the same island as the μ-LEED pattern. The superimposed red lines show the linear fit of the maxima intensity of each branch. (d) Representation of a positive doping effect on the Fermi level of graphene. (e) First Brillouin zone of graphene, where the high symmetry points are indicated. The crystallographic direction of underlying Rh(110) substrate is also specified. (f) μ-XPS C 1s spectra of oxygen-intercalated Gr/Rh(110) obtained in the same area as the μ-ARPES and μ-LEED using a photon energy of 400 eV. Experimental data are shown as white dots, and the background and the total fit are represented by the violet and black line, respectively. The spectrum can be deconvoluted into two components at binding energy 284.0 eV (blue) and 284.4 eV (red).

For a more comprehensive characterization of the Gr/O/Rh(110) nanostructures, the results presented and discussed above are complemented by LEEM, μ-LEED, μ-ARPES, and μ-XPS data. Figure 4a shows a BF-LEEM image taken at 10 eV on a region with isolated graphene islands over the metal surface. Apart from bare Rh(110) areas, two types of regions can be distinguished based on the image contrast. The μ-LEED pattern, acquired within the region marked by the orange circle in Figure 4a and shown in Figure 4b, exhibits three main sets of spots, as labeled in the figure. The outermost spots correspond to the graphene (encircled in green), while the Rh(110) spots (encircled in blue) form a rectangular array. Another important feature is highlighted by the dashed magenta rectangle, the interpretation of which will be given below.

First, as shown in Figure 4b, the graphene, in this case, is aligned (R0°) with the Rh[11̅0] direction in the reciprocal space, which corresponds to its alignment with the Rh[001] direction in real space. Interpretation of the LEEM image in Figure 4a requires a careful examination of the LEED pattern. Qualitatively, this pattern differs from that obtained on pristine aligned Gr/Rh(110).^18^ This suggests that the darker regions in the LEEM image are related to oxygen intercalation, while the other regions are not intercalated. Furthermore, the appearance of a new feature in the LEED pattern shown in Figure 4b, which is not present in the pattern reported for pristine Gr/Rh(110),^18^ reinforces this hypothesis. The diffuse path, highlighted by the dashed magenta rectangle, is aligned with the Rh[001] direction in the reciprocal space and shows a stronger intensity near the (0, 0) spot. Such a feature is consistent with the coexistence of different (1 × *n*) substrate reconstructions of Rh(110) under graphene, after the oxygen intercalation, in agreement with the STM measurements summarized and discussed in Figures 1 and2.

On the same island, where the μ-LEED shown in Figure 4b was realized, μ-ARPES and μ-XPS measurements were also performed. Using photon energy of 120 eV, Figure 4c shows the energy dispersion relation of oxygen-intercalated Gr/Rh(110), projected along the direction perpendicular to Γ–*K* of the graphene first Brillouin zone and centered on *K* (see the blue line in Figure 4e). Two linear branches are observed and can be attributed to the band structure of Gr/O/Rh(110) near the Fermi level. By fitting the intensity of each branch and plotting the positions of the maximum value as a function of the binding energy, two sets of points with linear dispersion are obtained. From the linear fits, the Fermi velocity (*v*_F_) and the Dirac point (*E*_D_) can be determined. Such a fitted result is represented by the two superimposed red lines, yielding an approximate value of *v*_F_ = 1.0 × 10^6^ ± 2 × 10^5^ m/s and *E*_D_ – *E*_F_ = 0.4 ± 0.1 eV. Details about the employed fitting procedure can be found in Figure S4. For completeness, the graphene band structure projected along other high symmetry directions of the graphene first Brillouin zone is shown in Figure S5. Similarly, using a photon energy of 400 eV, the C 1s core level spectrum of graphene is shown in Figure 4f. The spectrum can be deconvoluted into a dominant component centered at a binding energy of 284.0 eV and a minor component at 284.4 eV.

The estimated value for the Fermi velocity of graphene quasiparticles is in agreement with the theoretical and experimental values of a free-standing graphene monolayer.^44−46^ However, in the case of Gr/O/Rh(110), the Dirac point is above the Fermi level. In fact, the graphene layer shows a hole doping (see also the scheme in Figure 4d), indicating electron transfer from the graphene layer to the underlying oxygen atoms, which is probably due to the higher electronegativity of the latter. Moreover, the measured E_D_ agrees with the value reported by μ-ARPES for the oxygen-intercalated Gr/Ru(0001)^22^ system. However, it differs from the STS data obtained for oxygen intercalated on Gr/Rh(111)^23^ or Gr/Ru(0001).^47^ The absence of crossings of Rh(110) states with the graphene π band, which is present in as-grown graphene on Rh(110)^18^ suggests that, upon oxygen intercalation, the graphene layer is efficiently decoupled from Rh(110) and no further hybridization with the metal 4d states is expected, consistent with the analysis of graphene quasiparticle scattering results shown in Figure 3.

Compared to graphene on Rh(110),^18^ where the C 1s spectrum is described by two components with a binding energy of 284.9 and 284.4 eV, implying a significant interaction of graphene with the metal, the C 1s core level of graphene after oxygen intercalation below is characterized by a general shift toward lower binding energy, with a dominant peak at 284.0 eV and another one, with a more modest contribution to the C 1s line shape, at 284.4 eV. Such an energy shift is in qualitative agreement with the *p*-doping level of graphene, as demonstrated by the μ-ARPES data shown in Figure 4c. A similar core level shift of C 1s has been reported for oxygen-intercalated graphene/metal systems,^20,48^ and it is also consistent with other previously reported experimental results.^49^ Thus, the core level shift of the main component of the C 1s signal observed in the present case provides a solid fingerprint of oxygen intercalation under graphene. As shown in Figure 4f, the splitting of the C 1s spectra into two components proves the existence of two levels of interfacial interaction. The prominent peak centered at 284.0 eV could be assigned to the regions where graphene is decoupled from the substrate (IA and, possibly also, MRA). The smaller peak at 284.4 eV could correspond to the signal coming from bare Gr/Rh(110) regions without intercalation of oxygen atoms underneath, which, in reality, could have a very diluted oxygen layer intercalated. This would explain the absence of the component at 284.9 eV that is present in the Gr/Rh(110) system. This explanation is feasible since small patches (nanometer size) of bare Gr/Rh(110) can still be routinely found on a μ-scale area of oxygen-intercalated Gr/Rh(110) as evidenced during STM experiments (see, for example, Figures 3c and S6). However, a contribution to the component at 284.4 eV coming from MRA whose C atoms do not have oxygen atoms underneath them but are still lifted with respect to the metal substrate cannot be excluded.

## 4 Conclusions

By combining different imaging, spectroscopy, and diffraction techniques under UHV conditions, the structural and electronic properties of aperiodically modulated graphene monolayers have been investigated. Such a nanostructured system was realized by the intercalation of oxygen at the interface of graphene grown on Rh(110). Oxygen atoms under graphene form two types of superstructures with respect to Rh(110): the O-*c*(2 × 2), which does not involve any reconstruction of Rh(110), and a mixture of O-*c*(2 × 2*n*) phases with (1 × *n*) Rh(110) substrate reconstruction as missing rows, where *n* ranges from 4 to 12. The latter structure was the predominant one found in this oxygen-sandwiched system, and it is characterized by an aperiodic modulation of graphene over the surface due to the coexistence of different (1 × *n*) reconstructions within a narrow zone. The resulting aperiodic nanostructured graphene consists of two distinct regions, namely, the intercalated area (IA) and missing row area (MRA). Joint STM-based quasiparticle interference analysis and μ-ARPES and μ-XPS data obtained in the missing row reconstructed regions show that the graphene is efficiently decoupled from the Rh(110) substrate and exhibits the characteristic linear dispersion of graphene, albeit with *p*-doping. Furthermore, the experimental data do not support a significantly different chemical and electronic landscape on IA and MRA. These results could open an innovative method of graphene nanostructuring via the intercalation of species that induce surface reconstruction.

## Supporting Information Available

Continuity of the graphene layer across the missing row and the elongated Rh(110) islands (Figures S1–S2); intervalley scattering on IA and MRA (Figure S3); details about the fitting procedure used to analyze the ARPES data (Figure S4); μ-ARPES intensity plot along other high symmetry directions of graphene (Figure S5); coexistence of both pristine and intercalated Gr/Rh(110) areas (Figure S6) (PDF)
