# Supplementary material for: Aperiodic Modulation of Graphene Driven by Oxygen-Induced Reconstruction of Rh(110)
Source: J Phys Chem C Nanomater Interfaces. 2023 Aug 30;127(36):17930–8. doi: 10.1021/acs.jpcc.3c02643 (PMC10513088; doi:10.1021/acs.jpcc.3c02643)
Supplement: Supplementary file 1 — jp3c02643_si_001.pdf [file jp3c02643_si_001.pdf]

Supporting Information

Aperiodic Modulation of Graphene Driven by Oxygen Induced Reconstruction of Rh(110)

*Haojie Guo**[,1], *Mariano D. Jiménez-Sánchez*[1], *Enrique G. Michel*[1,2,3], *Antonio J. Martínez-Galera**[,2,4] *and José M. Gómez-Rodríguez*[1,2,3,†]

[1]Departamento de Física de la Materia Condensada, Universidad Autónoma de Madrid, E-28049 Madrid, Spain

[2]Instituto Nicolás Cabrera, Universidad Autónoma de Madrid, E-28049 Madrid, Spain

[3]Condensed Matter Physics Center (IFIMAC), Universidad Autónoma de Madrid, E-28049 Madrid, Spain

[4]Departamento de Física de Materiales, Universidad Autónoma de Madrid, E-28049 Madrid, Spain

***Corresponding authors:**

haojie.guo@uam.es (H. G.). Telephone: +34 91 497 69 11

antonio.galera@uam.es (A. J. M.- G.). Telephone: +34 91 497 24 50

S1. Graphene layer continuity along the missing row area

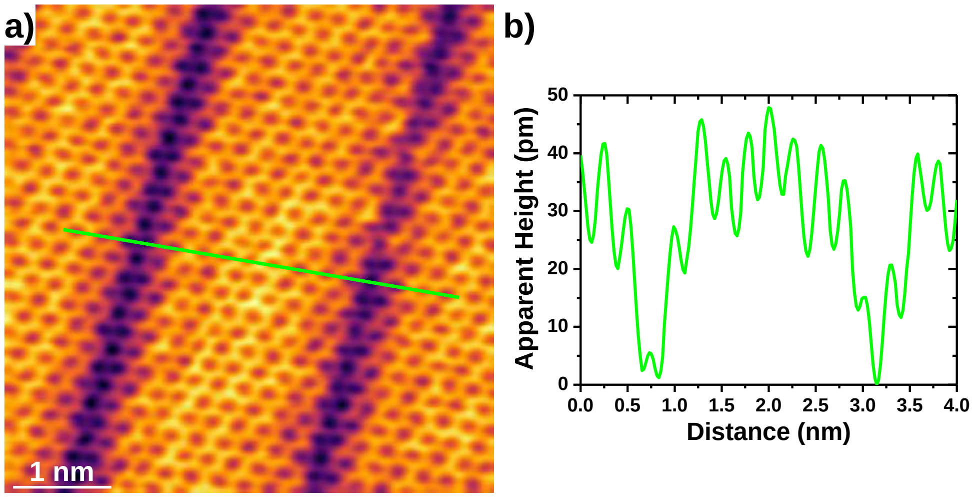

Figure S1. a) Atomic resolution STM image of Gr/O/Rh(110) highlighting the continuity of the graphene layer on the missing row area. Tunneling parameters: V_s = 0.13 V; I_t = 13.6 nA; size: 5 × 5 nm^2. b) Apparent height profile along the green line drawn in (a).

S2. Integrity of the graphene cover between islands of Rh(110) covered by oxygen

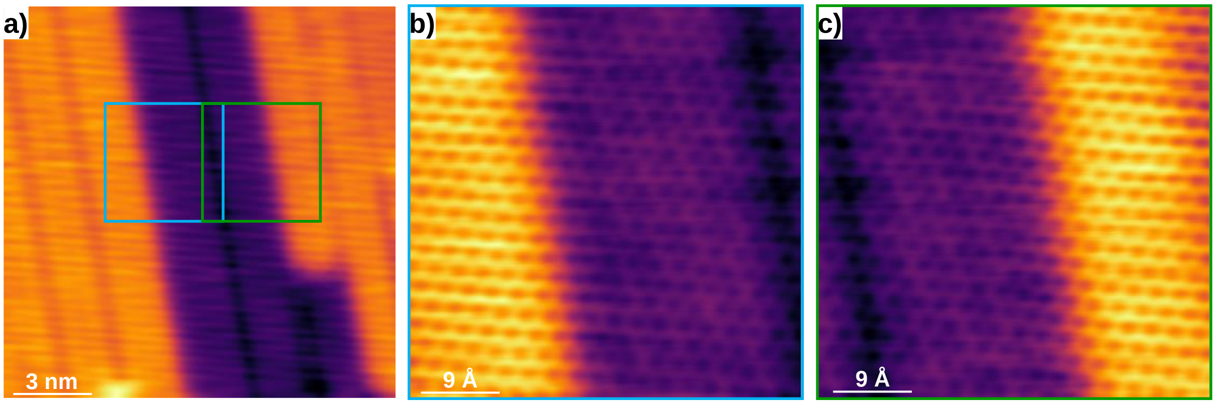

Figure S2. STM images indicating the continuity of graphene between islands of Rh(110) covered by oxygen. Tunneling parameters: a) $V_s = 2$ V; $I_t = 1$ nA; size: 15×15 nm^2. b) $V_s = 0.4$ V; $I_t = 3.1$ nA; size: 4.5×4.5 nm^2. c) $V_s = 0.4$ V; $I_t = 3.1$ nA; size: 4.5×4.5 nm^2.

S3. Intervalley scattering on IA and MRA

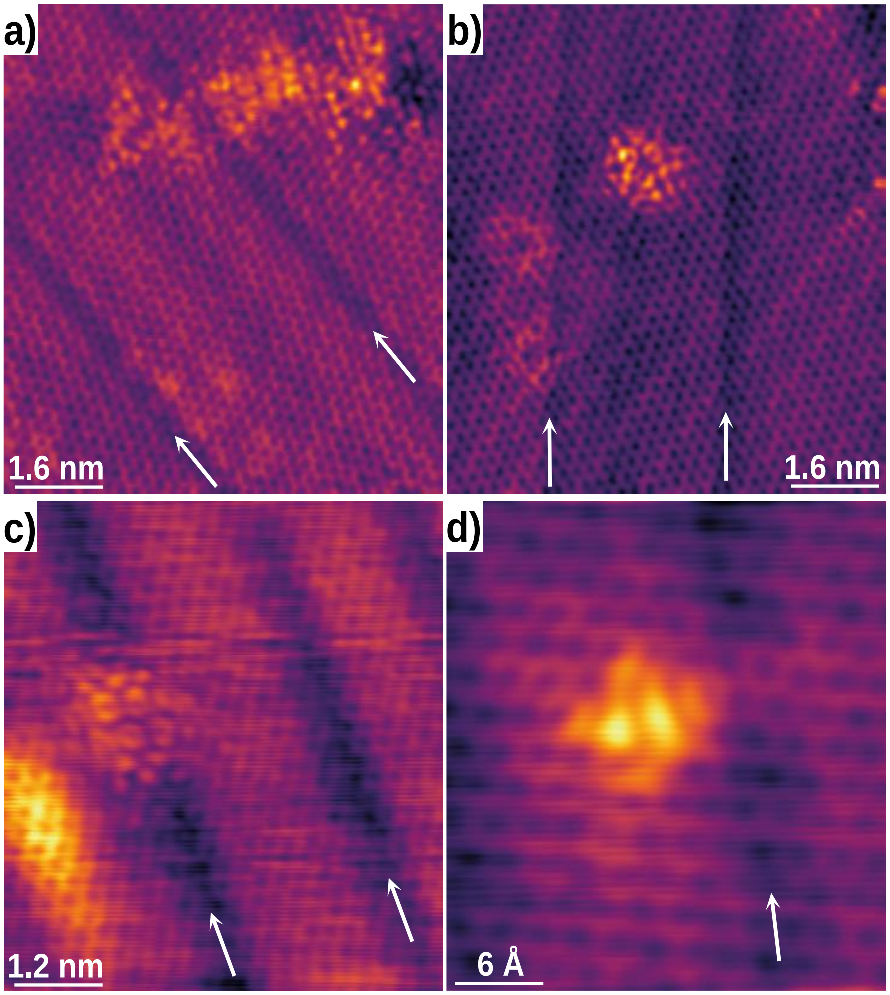

Figure S3. Additional STM images proving the existence of graphene quasiparticle interference, caused by different native defects present in graphene layer, on both intercalated and missing row area. White arrows indicate the $[1\bar{1}0]$ (missing row) direction. Tunneling parameters: a) $V_s = 60$ mV; $I_t = 6.1$ nA; size: 8×8 nm^2. b) $V_s = 0.15$ V; $I_t = 15.8$ nA; size: 8×8 nm^2. c) $V_s = -70$ mV; $I_t = 7.0$ nA; size: 6×6 nm^2. d) $V_s = 0.11$ V; $I_t = 3.0$ nA; size: 3×3 nm^2.

S4. The fitting procedure used to analyze the ARPES data

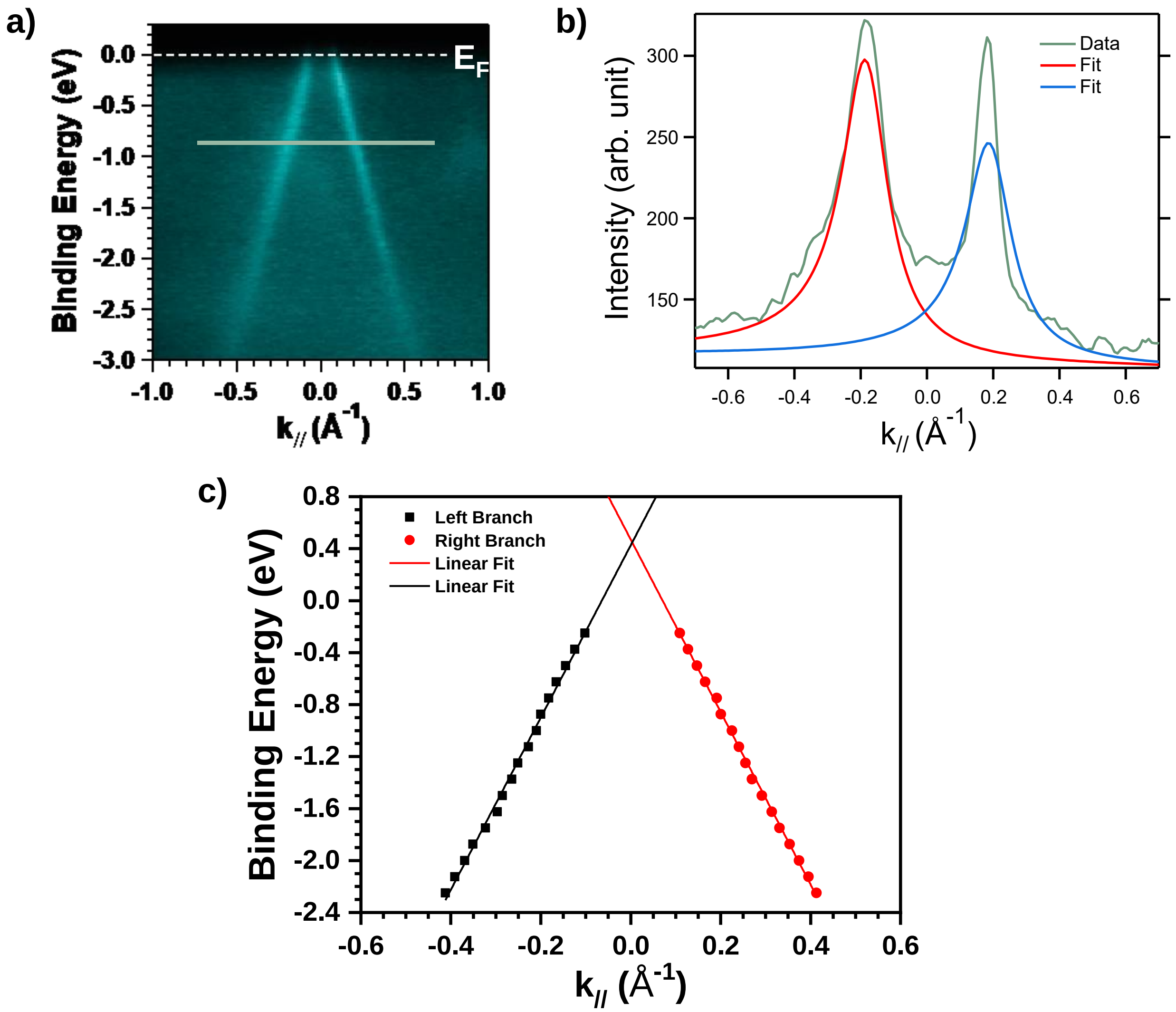

Figure S4. a) Same μ-ARPES intensity plot shown in Figure 4c without the fitting data superimposed. b) Green curve: Typical intensity plot corresponding to a certain binding energy (see the green line drawn in (a)). Red and blue curves: Fitted curves for both peaks. With these fittings, we can obtain the maxima intensity position ($k_{//}$) at the corresponding binding energy. We employed a Voight function to perform the fits. In general, the weighting of the Gaussian function was much lower than the Lorentzian function. c) Corresponding binding energy vs photoemission maxima intensity position graphs for both linear branches. Through a linear fit, we can derive the Fermi velocity (slope). Likewise, by interpolation from the fitting values, the Dirac point can also be estimated.

S5. μ-ARPES mapped along a perpendicular direction of Γ-K and Γ-K-M-Γ directions

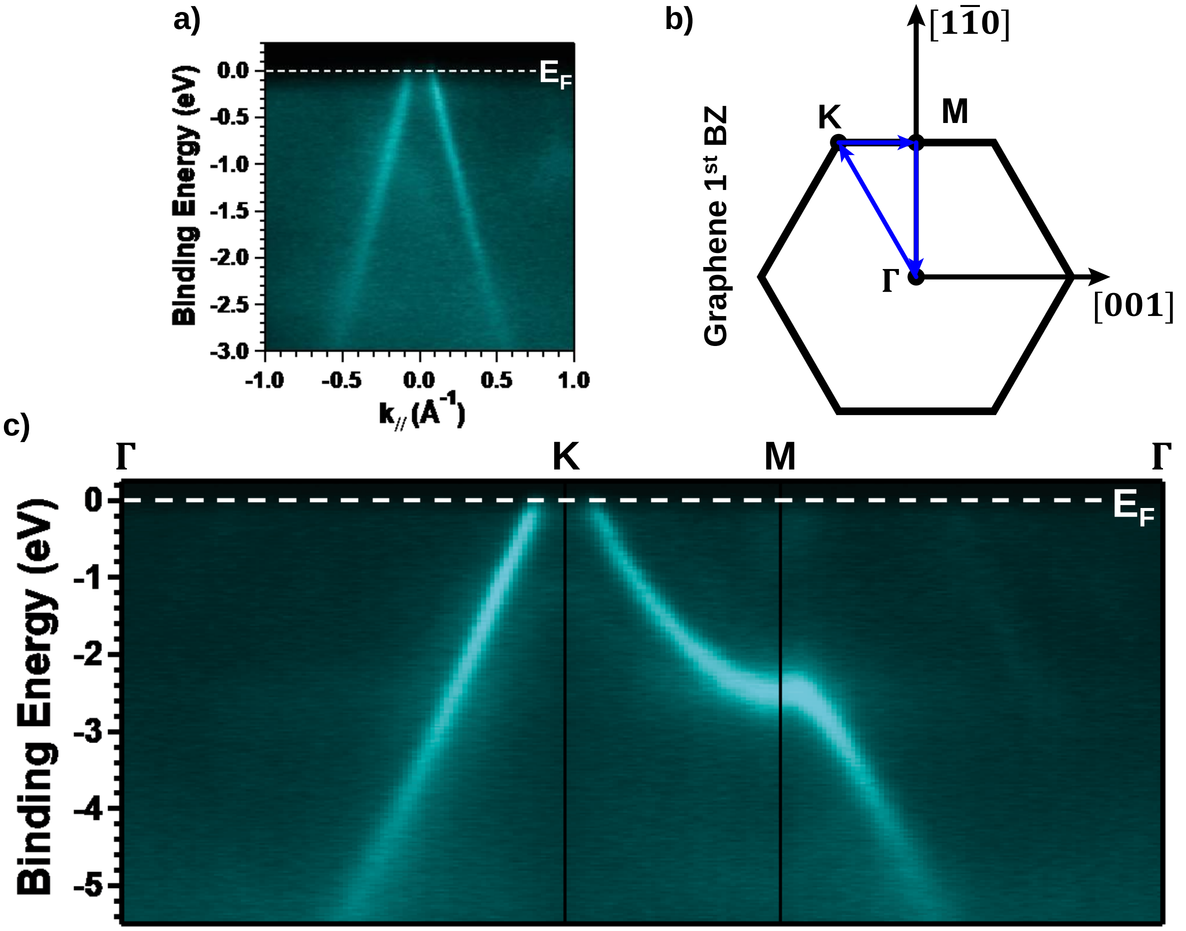

Figure S5. a) Same μ-ARPES intensity plot shown in Figure 4c without the fitting data superimposed. b) Schematic representation of the graphene First Brillouin Zone where the high symmetry points are labelled. The crystallographic directions of underlying Rh(110) substrate are also specified. c) μ-ARPES energy dispersion relation of Gr/O/Rh(110) measured along the three high symmetric direction of graphene in the reciprocal space, as indicated by the blue triangle in (b).

S6: Coexistence of oxygen intercalated and non-intercalated regions of Gr/Rh(110)

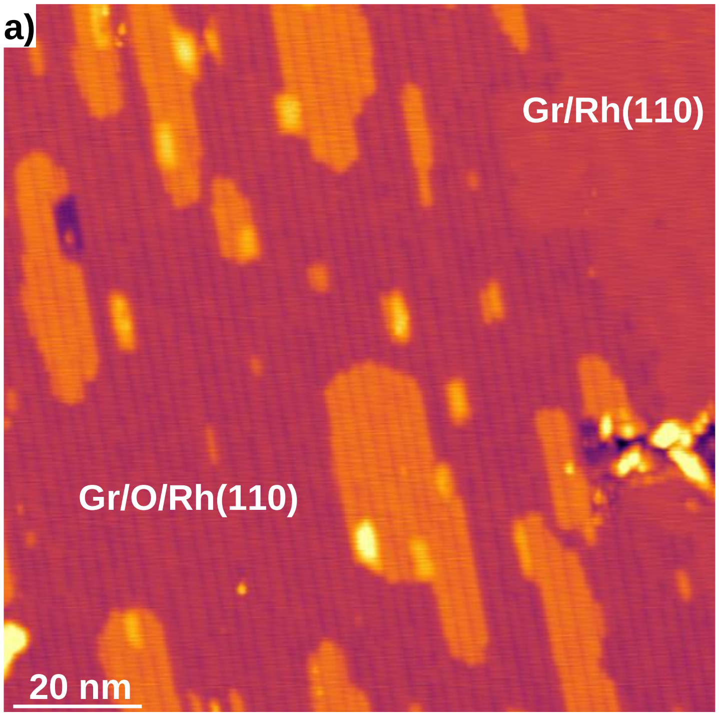

Figure S6. Coexistence of small areas of pristine Gr/Rh(110) with oxygen intercalated Gr/Rh(110). Tunneling parameters: a) $V_s = 2$ V; $I_t = 0.3$ nA; size: 100×100 nm^2.
